# *MLPK* function is not required for self-incompatibility in the *S*^*29*^ haplotype of *Brassica rapa* L.

**DOI:** 10.1007/s00497-023-00463-w

**Published:** 2023-04-26

**Authors:** Mayu Ohata, Yoshinobu Takada, Yui Sato, Takumi Okamoto, Kohji Murase, Seiji Takayama, Go Suzuki, Masao Watanabe

**Affiliations:** 1 Graduate School of Life Sciences, Tohoku University, Sendai, 980-8577 Japan; 2 Department of Applied Biological Chemistry, Graduate School of Agricultural and Life Sciences, The University of Tokyo, Tokyo, 113-8657 Japan; 3 Division of Natural Science, Osaka Kyoiku University, Kashiwara, 582-8582 Japan; 4 Present Address: Department of Pharmaceutical Sciences, University of Shizuoka, Shizuoka, 422-8526 Japan

**Keywords:** *Brassica rapa*, MLPK, Pollen-stigma interaction, Self-incompatibility, *S* haplotype, SRK

## Abstract

**Key message:**

*S*^*29*^ haplotype does not require the *MLPK* function for self-incompatibility in *Brassica rapa*.

**Abstract:**

Self-incompatibility (SI) in Brassicaceae is regulated by the self-recognition mechanism, which is based on the *S*-haplotype-specific direct interaction of the pollen-derived ligand, SP11/SCR, and the stigma-side receptor, SRK. *M* locus protein kinase (MLPK) is known to be one of the positive effectors of the SI response. MLPK directly interacts with SRK, and is phosphorylated by SRK in *Brassica rapa*. In Brassicaceae, MLPK was demonstrated to be essential for SI in *B. rapa* and *Brassica napus*, whereas it is not essential for SI in *Arabidopsis thaliana* (with introduced *SRK* and *SP11/SCR* from related SI species). Little is known about what determines the need for MLPK in SI of Brassicaceae. In this study, we investigated the relationship between *S*-haplotype diversity and MLPK function by analyzing the SI phenotypes of different *S* haplotypes in a *mlpk*/*mlpk* mutant background. The results have clarified that in *B. rapa*, all the *S* haplotypes except the *S*^*29*^ we tested need the MLPK function, but the *S*^*29*^ haplotype does not require MLPK for the SI*.* Comparative analysis of MLPK-dependent and MLPK-independent *S* haplotype might provide new insight into the evolution of *S*-haplotype diversity and the molecular mechanism of SI in Brassicaceae.

**Supplementary Information:**

The online version contains supplementary material available at 10.1007/s00497-023-00463-w.

## Introduction

Self-incompatibility (SI) is controlled by a single locus, called the *S* locus, with highly polymorphic multiple alleles (Bateman 1955). In Brassicaceae, the *S*-locus region contains two genes, stigma and pollen recognition determinant genes, *S receptor kinase* (*SRK*) and *S-locus protein 11* (*SP11*, also called *SCR*), respectively (Stein et al. 1991; Takasaki et al. 2000; Suzuki et al. 1999; Schopfer et al. 1999; Takayama et al. 2000; reviewed in Watanabe et al. 2012; Fujii and Takayama 2018; Abhinandan et al. 2021). *S*-haplotype-specific direct interaction of SRK and SP11 causes phosphorylation of SRK and induces self-pollen rejection (Takayama et al. 2001; Shimosato et al. 2007; Murase et al. 2020). Because the *SRK* and *SP11* genes are inherited as a single segregational unit, *S* alleles are termed *S* haplotypes (Nasrallah and Nasrallah 1993). *S*-haplotype diversity is determined by sequence polymorphism of *SRK* and *SP11* genes in the *S* locus and over 100 have been identified (*S*^*1*^, *S*^*2*^, *S*^*3*^, …, *S*^*n*^) in the genus *Brassica* (Nou et al. 1993; Sakamoto and Nishio 2001; Watanabe et al. 2000; Ockendon 1982; Charlesworth et al. 2003; Paetsch et al. 2006), and over 30 in *Raphanus* (Kim and Kim 2019; Fukushima et al. 2021). In *Brassica*, *S* haplotypes are classified into two classes (class-I and -II) based on the sequence similarity of the extracellular domain encoded by *SRK* (and its homologous gene termed *SLG*). The pollen-side SI phenotype is almost co-dominant between class-I *S* haplotypes, and the stigma-side is also co-dominant with a few exceptions (Hatakeyama et al. 1998b). On the other hand, class-II *S* haplotypes are generally recessive to class-I *S* haplotypes in pollen, but the two classes are co-dominant in the stigma (Hatakeyama et al. 1998b). Diploid plants carrying two co-dominant *S* haplotypes exhibit SI specificity of both *S* haplotypes encoded by the parental genome. The regulation of *SP11* gene expression controls this dominance relationship on the pollen side through epigenetic mechanisms, including small RNA-mediated DNA methylation (Shiba et al. 2002; Kakizaki et al. 2003; Tarutani et al. 2010; Yasuda et al. 2016).

Studies of the molecular mechanism of SI in Brassicaceae have been conducted mainly in *Brassica* species (*Brassica rapa*, *Brassica oleracea*, *Brassica napus*). However, in *Arabidopsis thaliana*, a naturally self-compatible species, it has been reported that SI can be imparted by introducing *SRK* and *SP11* genes of closely related SI species (*Arabidopsis lyrata*, *Arabidopsis halleri*) (Nasrallah et al. 2002; Zhang et al. 2019; Fujii et al. 2020). In addition, it has been reported that a change from self-compatibility (SC) to SI occurs by restoring the inversion of the *SP11* gene in *A. thaliana* (Tsuchimatsu et al. 2010). This suggests that the components required for the SI reaction are common to *Brassica* and *Arabidopsis* species.

In the *Brassica* SI system, downstream signaling pathways and the target of SRK leading to self-pollen rejection are becoming better understood. The *M*-locus protein kinase (MLPK), which was isolated by positional cloning of the *M* locus of the self-compatible *B. rapa* variety ‘*yellow sarson’*, is an essential positive regulator of the SI response (Murase et al. 2004). MLPK belongs to the receptor-like cytoplasmic kinase family. Biochemical analysis revealed that MLPK is a membrane-bound kinase present in the cell membrane fraction of the stigma, the N-terminal myristoylation motif is involved in membrane localization (Murase et al. 2004), and MLPK directly interacts with and is phosphorylated by SRK (Kakita et al. 2007). Because MLPK-deficient plants exhibit a completely SC phenotype in *B. rapa* and *B. napus*, MLPK is considered to be an essential protein that positively regulates the self-incompatibility signaling system in *Brassica* (Murase et al. 2004; Chen et al. 2019). On the other hand, it has been reported that mutation of *APK1b*, the gene in *A. thaliana* that shares the highest similarity to *MLPK,* does not affect the SI response of self-incompatible *SRK*/*SP11* transgenic *A. thaliana* (Kakita et al. 2007; Kitashiba et al. 2011), and *APK1b* has been shown to be involved in light-induced stomatal opening in *A. thaliana* (Elhaddad et al. 2014). According to the analysis by Azibi et al. (2020), there are 3 copies of *MLPK* in the genus *Brassica,* which have arisen by whole-genome triplication (WGT), and in the phylogenetic analysis, the gene closest to *APK1b* in *B. rapa* is one of the paralogues of *MLPK*, not *MLPK* itself. Thus, the recruitment of MLPK in SI signaling in *Brassica* may be the result of neo-functionalization of the duplicated genes, which occurred after the WGT in the origin of *Brassica* species (Azibi et al. 2020).

In this study, we investigated the genetic association of MLPK with the SI signaling of different *S* haplotypes in *B. rapa*. We found that the* S*^*29*^ haplotype of *B. rapa* does not require MLPK functionality in SI, unlike other *S* haplotypes.

## Materials and methods

### Plant materials and test pollination

Plant materials used in this study are listed in Supplementary Table S1. As the *mlpk/mlpk* mutant donor line, we used *S*^*8*^*/S*^*8*^, *mlpk/mlpk* (hereafter referred to as *S*^*8*^*S*^*8*^, *mm*) from Murase et al. (2004). The 11 *S* homozygous lines of *B. rapa* used in the present experiment were selected from those established by Nou et al. (1993) containing two different populations, from Oguni in Japan and from Balcesme in Turkey. In addition to these 11 lines, one *S* homozygous line (*S*^*60*^), which was derived from a Japanese commercial hybrid variety (cv. Osome, Takii & Co., Ltd, Takasaki et al 1999), was also used in this study. All *S* homozygous lines have been confirmed to exhibit the self-incompatible phenotype (Nou et al. 1993; Takasaki et al 1999). Pollination phenotype was determined by test cross as described in Takada et al. (2017). Pollinated stigmas were stained with aniline blue and observed by UV fluorescence microscopy (Zeiss Axio Imager A2, Kho and Bear 1968). The degree of compatibility (compatibility score; CS) in each test pollination was scored on a five-point scale based on pollen tube penetration as follows: (CS = 5) penetration of more than 10 pollen tubes into the style; (CS = 4) penetration of 1–10 pollen tubes into the style; (CS = 3) penetration of pollen tubes into papilla cells but not into the style; (CS = 2) germination of pollen but no pollen-tube penetration into papilla cells; (CS = 1) no germination of pollen. Average scores less than 3 were defined as incompatible, and scores 3 and above as compatible (Takada et al. 2017; 2021). Stigmas from at least three flowers for each cross combination were tested and this was replicated on at least five different dates.

### Determination of the *S* haplotype and *MLPK* genotype

The *S* haplotype of each plant was determined by polymerase chain reaction (PCR). Total DNA was extracted from young leaf tissue of *B. rapa* using DNeasy plant mini kit (Qiagen). PCR was performed using Ex*Taq* DNA polymerase (Takara Bio). To determine the *S* haplotype of each plant, class I/class II-specific PCR primers for amplification of *SLG* were used, as described in Nishio et al. (1996). The each *SP11* genes were amplified by using *S*-haplotype-specific *SP11* primer sets (Supplementary Table S2). For genotyping of *MLPK*, the functional *MLPK* allele was specifically amplified using the PCR primers wtMLPK-F and wtmMLPK-R, and the mutated *mlpk* allele was specifically amplified using the mMLPK-F and wtmMLPK-R primers (Takada et al. 2013). The PCR product was subjected to electrophoresis on a 1% agarose gel. For isolation of *MLPK* genomic sequences from the *S*^*29*^ haplotype, PCR amplification using a primer pair MLPKF and MLPKR was performed, and amplified fragments were cloned into pTAC2 vector (Biodynamics Laboratory Inc.). The nucleotide sequence was determined with a 3500 Genetic Analyzer using Big Dye Terminator version 3.1 Cycle Sequencing Kit (Applied Biosystems).

### Sequence and multiple alignment analysis

Accession numbers of SRK sequences used in amino acid alignment analysis are listed in Supplementary Table S3. GENETYX version 13 software package (GENETYX Corp.) was used for the sequence comparison and alignment.

## Results and discussion

**Fig. 1:**
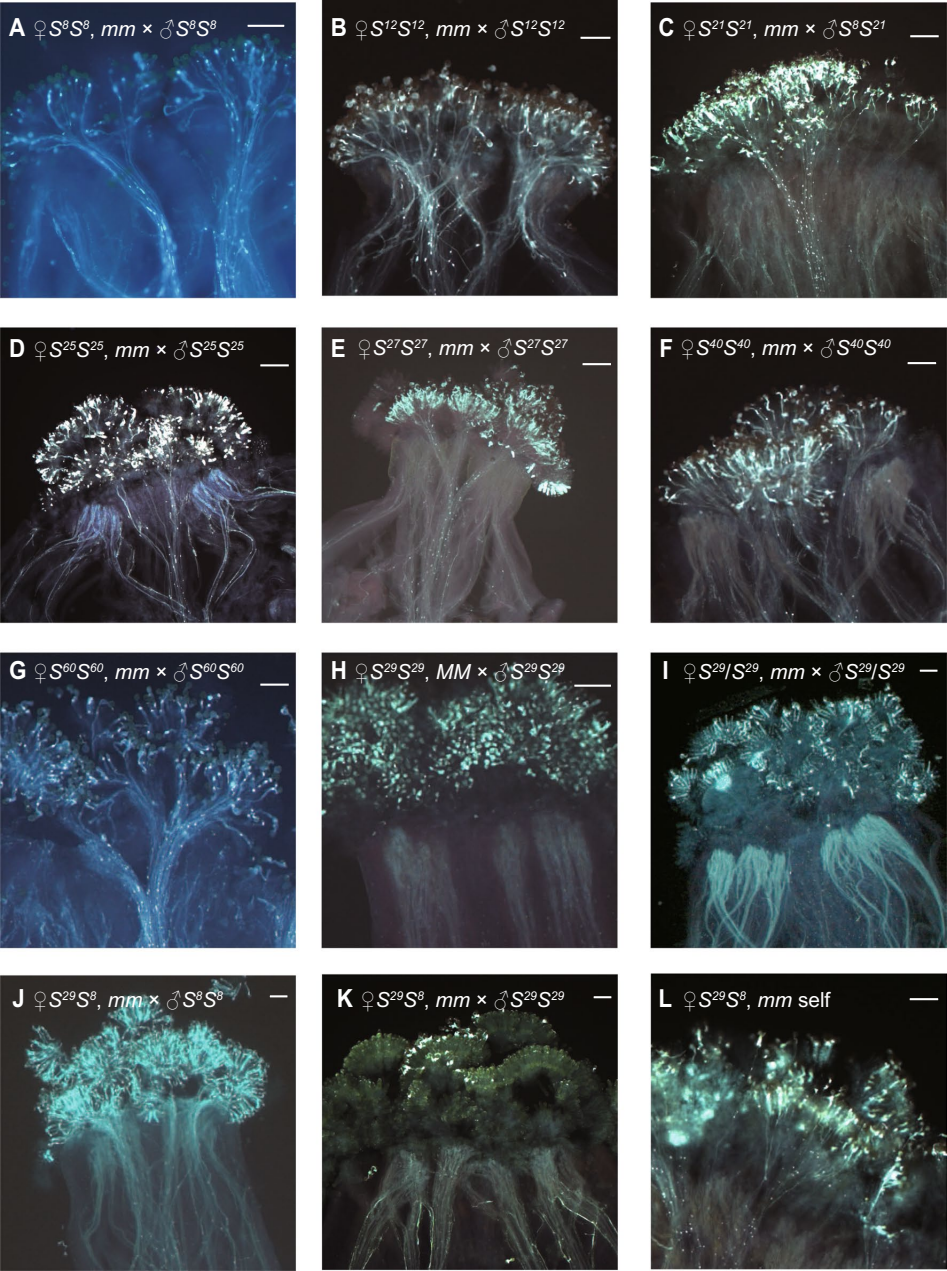
Representative photographs of test crosses. Photographs were obtained by UV fluorescence microscopy (a-l). **a** Cross pollination of *S*^*8*^*S*^*8*^, *mm* stigma with *S*^*8*^*S*^*8*^ tester pollen (♀*S*^*8*^*S*^*8*^, *mm* × ♂*S*^*8*^*S*^*8*^). **b** ♀*S*^*12*^*S*^*12*^, *mm* × ♂*S*^*12*^*S*^*12*^, **c** ♀*S*^*21*^*S*^*21*^*, mm* × ♂*S*^*8*^*S*^*21*^, **d** ♀*S*^*25*^*S*^*25*^, *mm* × ♂*S*^*25*^*S*^*25*^, **e** ♀*S*^*27*^*S*^*27*^*, mm* × ♂*S*^*27*^*S*^*27*^, **f** ♀*S*^*40*^*S*^*40*^*, mm* × ♂*S*^*40*^*S*^*40*^, **g** ♀*S*^*60*^*S*^*60*^*, mm* × ♂*S*^*60*^*S*^*60*^, **h** ♀*S*^*29*^*S*^*29*^*, MM* × ♂*S*^*29*^*S*^*29*^*,* **i**) ♀*S*^*29*^*S*^*29*^*, mm* × ♂*S*^*29*^*S*^*29*^, **j** ♀*S*^*29*^*S*^*8*^*, mm* × ♂*S*^*8*^*S*^*8*^, **k** ♀*S*^*29*^*S*^*8*^*, mm* × ♂*S*^*29*^*S*^*29*^, (l) *S*^*29*^*S*^*8*^*, mm* self-pollination. *MLPK* genotype of pollen donors was *MM* in cross-pollinations and *mm* in self-pollinations. Scale bars show 100 μm

The breakdown of SI with *S*^*8*^ haplotype in *mm* mutant background has been reported previously (Murase et al. 2004; Fukai et al. 2001). We tested the SC phenotype of *S*^*8*^*S*^*8*^, *mm* (*S*^*8*^*/S*^*8*^, *mlpk/mlpk*) homozygous plants, established by Murase et al. (2004). Self-pollination of 11 *S*^*8*^*S*^*8*^, *mm* homozygous plants showed SC phenotype, and the test cross between the stigma of 34 *S*^*8*^*S*^*8*^, *mm* homozygous plants and the pollen from *S*^*8*^*S*^*8*^, *MM* tester line resulted in compatible pollination (Table 1, Fig. 1A). To analyze details of the genetic relationship between *S* haplotype diversity and MLPK function in the SI of *Brassica*, we established *mm* mutant lines possessing the different *S*-haplotype backgrounds by crossing *S*^*8*^*S*^*8*^, *mm* homozygous SC plants with 7 class-I and 4 class-II *S* haplotypes (Table 1, 2, Supplementary Table S1). Each *S* haplotype and *MM* homozygous plant was crossed with *S*^*8*^*S*^*8*^, *mm* plants. The *mm* plants in each *S* haplotype were selected from the F_2_ segregating population. The obtained *mm* homozygous plants were test crossed with the pollen from *S* homozygous tester lines (Supplementary Table S1). The *S*^*12*^ and *S*^*24*^ haplotypes have the same SI recognition identity but originated from different locations (*S*^*12*^ from Japan, *S*^*24*^ from Turkey, Nou et al. 1993; Matsushita et al. 1996; Takada et al. 2017). In the class-I *S* haplotypes (*S*^*12*^, *S*^*24*^, *S*^*21*^, *S*^*25*^, *S*^*27*^, *S*^*35*^, *S*^*37*^, and *S*^*45*^) examined in this study, all individuals with the *mm* homozygous background showed compatibility phenotype on the stigma side, indicating that MLPK is essential for SI in these class-I haplotypes (Table 1, Fig. 1B-E). We observed fully germinated and penetrated pollen tubes in each compatible cross (Fig. 1B-E).

**Table 1:** Pollination phenotype of *mlpk* mutants in different class-I *S* haplotypes

Genotype of parents			Phenotype	
Female	Male	*n*	I	C
*S*^*8*^*S*^*8*^*MM*	self	*6*	6	0
*S*^*8*^*S*^*8*^*mm*	self	11	0	11
	*S*^*8*^*S*^*8*^	34	0	34
*S*^*12*^*S*^*12*^*mm*	*S*^*12*^*S*^*12*^	4	0	6
	*S*^*8*^*S*^*8*^	4	0	6
*S*^*24*^*S*^*24*^*mm*	*S*^*24*^*S*^*24*^	1	0	1
	*S*^*8*^*S*^*8*^	1	0	1
*S*^*25*^*S*^*25*^*mm*	*S*^*25*^*S*^*25*^	2	0	2
	*S*^*8*^*S*^*8*^	2	0	2
*S*^*21*^*S*^*21*^*mm*	*S*^*21*^*S*^*21*^	4	0	4
	*S*^*8*^*S*^*8*^	4	0	4
*S*^*27*^*S*^*27*^*mm*	*S*^*27*^*S*^*27*^	5	0	5
	*S*^*8*^*S*^*8*^	5	0	5
*S*^*37*^*S*^*37*^*mm*	*S*^*37*^*S*^*37*^	2	0	2
	*S*^*8*^*S*^*8*^	2	0	2
*S*^*45*^*S*^*45*^*mm*	*S*^*45*^*S*^*45*^	3	0	3
	*S*^*8*^*S*^*8*^	3	0	3

The number of plants showing incompatibility or compatibility phenotype is represented. *I*: Incompatible; *C*: Compatible

**Table 2:** Pollination phenotype of *mlpk* mutants in different class-II *S* haplotypes

Genotype of parents			Phenotype	
Female	Male	*n*	I	C
*S*^*29*^*S*^*29*^*mm*	*S*^*29*^*S*^*29*^	8	8	0
	*S*^*8*^*S*^*8*^	8	0	8
*S*^*40*^*S*^*40*^*mm*	*S*^*40*^*S*^*40*^	6	0	6
*S*^*44*^*S*^*44*^*mm*	*S*^*44*^*S*^*44*^	7	0	7
*S*^*60*^*S*^*60*^*mm*	*S*^*60*^*S*^*60*^	4	0	4

The number of plants showing incompatibility or compatibility phenotype is represented. *I*: Incompatible; *C*: Compatible

Among 4 class-II *S* haplotypes (*S*^*29*^, *S*^*40*^, *S*^*44*^, and *S*^*60*^) reported in Kakizaki et al. (2003), *S*^*40*^, *S*^*44*^, and *S*^*60*^ haplotypes with *mm* exhibited SC phenotype (compatibility with the pollen from plants possessing the same *S* haplotype) as in the class-I *S*-haplotypes (Table 2, Fig. 1F, G), indicating that the function of *MLPK* is required for SI recognition and reaction in these 3 class-II *S* haplotypes (*S*^*40*^, *S*^*44*^, and *S*^*60*^). However, the *S*^*29*^ haplotype with *mm* unexpectedly exhibited the SI reaction, which could not be explained by the current theory of *Brassica* self-incompatibility (Table 2, Fig. 1H, I). The stigma of 8 *mlpk* mutant plants with the *S*^*29*^ allele (*S*^*29*^*S*^*29*^, *mm*) showed SI phenotype and incompatibility with the pollen from *S*^*29*^*S*^*29*^ tester plants (Table 2, Fig. 1I). We could not distinguish the SI phenotype of *S*^*29*^*S*^*29*^, *mm* plants and the SI of wild type *S*^*29*^*S*^*29*^, *MM* plant. This result is the first detection of an MLPK-independent SI phenotype in the genus *Brassica*.

To further improve the experiments in Table 1 with limited plant number, and to test the MLPK function in *S*-heterozygous plants, we selected *mm* mutant plants from lines heterozygous for *S*^*8*^ haplotype and 7 other *S* haplotypes (*S*^*24*^, *S*^*25*^* S*^*45*^, *S*^*29*^, *S*^*40*^, *S*^*44*^ and *S*^*60*^) and checked the pollination phenotype (Table 3). The stigma-side dominant relationship of all *S*-haplotype combinations used in this study have been reported as co-dominant, except *S*^*8*^*S*^*60*^ heterozygous plants (Hatakeyama et al. 1998b). The stigma-side co-dominant relationship of *S*^*8*^*S*^*60*^ heterozygous plants was determined in this study (data not shown). All the combinations except *S*^*8*^*S*^*29*^ exhibited compatible phenotype with the pollen from its own *S* haplotype (Table 3). When we checked the pollination phenotype of 22 *S*^*8*^*S*^*29*^ heterozygous plants (*S*^*8*^*S*^*29*^, *mm*), all 22 plants showed compatibility with the pollen from *S*^*8*^*S*^*8*^ plants and incompatibility with the pollen from *S*^*29*^*S*^*29*^ plants (Table 3, Fig. 1J-L). It is interesting that the SI signal in SRK^29^ appears to be retained although the SRK^8^-mediated signal transduction appears to have been lost by *mlpk* mutation in the *S*^*8*^*S*^*29*^, *mm* plants.

**Table 3:** Analysis of MLPK dependence and stigma-side dominance relationship

	Genotype of parents		Stigma-side dominance relationship		Phenotype	
Class	Female	Male		*n*	I	C
I	*S*^*8*^*S*^*24*^*mm*	*S*^*24*^*S*^*24*^	*S*^*8*^ = *S*^*24*^	6	0	6
		*S*^*8*^*S*^*8*^		6	0	6
I	*S*^*8*^*S*^*25*^*mm*	*S*^*25*^*S*^*25*^	*S*^*8*^ = *S*^*25*^	6	0	6
		*S*^*8*^*S*^*8*^		6	0	6
I	*S*^*8*^*S*^*45*^*mm*	*S*^*45*^*S*^*45*^	*S*^*8*^ = *S*^*45*^	6	0	6
		*S*^*8*^*S*^*8*^		6	0	6
II	*S*^*8*^*S*^*29*^*mm*	*S*^*29*^*S*^*29*^	*S*^*8*^ = *S*^*29*^	22	22	0
		*S*^*8*^*S*^*8*^		22	0	22
II	*S*^*8*^*S*^*40*^*mm*	*S*^*40*^*S*^*40*^	*S*^*8*^ = *S*^*40*^	6	0	6
		*S*^*8*^*S*^*8*^		6	0	6
II	*S*^*8*^*S*^*44*^*mm*	*S*^*44*^*S*^*44*^	*S*^*8*^ = *S*^*44*^	9	0	9
		*S*^*8*^*S*^*8*^		9	0	9
II	*S*^*8*^*S*^*60*^*mm*	*S*^*60*^*S*^*60*^	*S*^*8*^ = *S*^60^*	6	0	6
		*S*^*8*^*S*^*8*^		6	0	6

The number of plants showing incompatibility or compatibility phenotype is represented. *I*: Incompatible; *C*: Compatible. *Class*: Class of tested *S*: Haplotype. Stigma-side dominance relationship is co-dominant in all combinations (Hatakeyama et al. 1998b; *, The dominance relationship of *S*^*8*^*S*^*60*^ combination was determined in this study

**Fig. 2:**
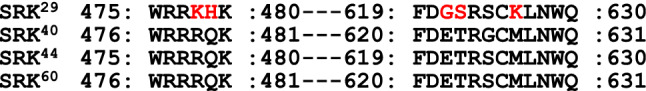
Sequence alignment of *Brassica* SRK cytoplasmic region. The SRK^29^-specific amino acid sites are shown in red

Moreover, the complete genetic linkage between the *S* locus with *S*^*29*^ haplotype and the MLPK-independent SI phenotype suggests that the *S*^*29*^ haplotype does not require MLPK for the SP11/SRK-based SI signal transduction, rather than the existence of other genetic factor(s), which can complement the *mlpk* mutation, in the vicinity of *S* locus of the *S*^*29*^ haplotype (Table 2, 3). One of the three duplicated *MLPK-*like genes, *A07p21240.2.BraZ1*(*B. rapa* Z1 ver. 2, Istace et al. 2021), is located on chromosome A07 where the *S*-locus resides, but the two loci are far apart (Azibi et al 2020). Therefore, it is unlikely that the *A07p21240.2.BraZ1* gene on chromosome A07 of the *S*^*29*^ haplotype has overlapping functions with *MLPK*. It is commonly inferred that the MLPK dependence of the SI mechanism is associated with direct or indirect binding of MLPK to the SRK or SP11-SRK complex, phosphorylation of MLPK by SRK, or both. Here, we isolated and sequenced the *MLPK* gene from the *S*^*29*^ haplotypes, and we could not find any difference at the nucleotide level between *S*^*29*^ and *S*^*8*^ haplotypes (data not shown, the *MLPK* sequence in the *S*^*8*^ haplotype has been reported in Murase et al. 2004). To clarify the specific amino acid sites of SRK^29^, especially in the intracellular region (considered to bind with MLPK), we compared amino acids sequences of SRK from class-II *S* haplotypes (Fig. 2, Supplementary Fig. S1). The genetic diversity of *SRK* gene in class-II *S* haplotypes is reported to be lower than class-I *S* haplotypes (Hatakeyama et al 1998a). The result showed that only five amino acids are specific to SRK^29^, compared with three other SRKs (SRK^40^, SRK^44^, and SRK^60^, Fig. 2, Supplementary Fig. S1). Further biochemical and genetic experiments are needed, but these five amino acids may determine MLPK dependence in *B. rapa*.

In this study, we revealed that all *S*-haplotypes except the *S*^*29*^ haplotype have a MLPK-dependent SI system. Furthermore, we also showed that the *S*^*29*^ haplotype does not require MLPK for the SI system. Because there have been reports that MLPK is a co-factor for SRK kinase function, it is likely that SRK alone transduces the phosphorylation signal to the downstream target of SI response in the *S*^*29*^ haplotype (Murase et al. 2004; Kakita et al. 2007; Chen et al. 2019). The *S*^*29*^ haplotype of *B. rapa* has been characterized as the most recessive in the pollen-side dominance relationship (Hatakeyama et al. 1998b; Kakizaki et al. 2003). It is interesting to consider whether there is any relationship between the dominance relationship and the discovery of the MLPK-independent SI system in *B. rapa*. Two hypotheses can be proposed: one is that the *S*^*29*^ haplotype is the ancestral *S* haplotype in *Brassica,* which does not need MLPK, as in *Arabidopsis*, and the other is that the *S*^*29*^ haplotype has lost its MLPK dependence after it had previously been acquired. In either case, this study reveals that the SI signaling pathway does not require MLPK in the *S*^*29*^ haplotype of *B. rapa*, raising the further question of why many *S* haplotypes in *B. rapa* need MLPK. After the *Arabidopsis*-*Brassica* diversification and the significant reduction in the number of ancient *S* haplotypes, the *S* haplotype of *Brassica* is thought to have rapidly increased in diversity in a very limited time (Kusaba et al. 2001; Edh et al. 2009). It is interesting to consider that the acquisition of MLPK, as the intracellular co-receptor with SRK in the SI signaling pathway, contributed to this rapid spread of the *S* haplotype.

### Author contribution statement

MO, YT and MW designed the experiments. MO, YT, YS and TO performed the experimental work and collected data. YT, KM, ST, GS and MW wrote the manuscript. All authors read and approved the manuscript.

## Supplementary Information

Below is the link to the electronic supplementary material.

Supplementary file1 (DOCX 45 KB)

Supplementary file2 (XLSX 28 KB)
