# Supplementary material for: MLPK function is not required for self-incompatibility in the S29 haplotype of Brassica rapa L
Source: Plant Reprod. 2023 Apr 26;36(3):255–62. doi: 10.1007/s00497-023-00463-w (PMC10363064; doi:10.1007/s00497-023-00463-w)
Supplement: Supplementary file 1 — Supplementary file1 (DOCX 45 KB) [file 497_2023_463_MOESM1_ESM.docx]

**SRK29 454:IGWSIGVSVMLILSVI---VFCFWRRKHKQAKADATPIVG---NQVLMNEVVLPRKKRNF 507**

**SRK40 455:................---.......RQ............---..............IH. 508**

**SRK44 454:................---.......RQ............---................. 507**

**SRK60 455:................---.......RQ............---...............I. 508**

**SRK8 445:.SLIV....V.L.LLLLLIM..L.K..QNR...M..S..NQQR..NVLMNTMTQSN..QL 504**

**SRK12 448:.SLIV....-.L.LIM----..L.K..KNR...S..S.DNQQR..NVLMNGMTQSN..QL 502**

**SRK21 449:.TLIV..GL-.FIMIV----..L.K..Q.RG.EI..S..NRQR.HDVLINGMILSS..QL 503**

**SRK25 433:.SLAV....-.L.LI.----..V.K..Q.....K..S.ANRQR..N.PMNGMVLST..E. 487**

**SRK35 436:VSLIV....V.L.LLL--IG..L.K..QNR...M..S..NQQR..NVLMNTMTQSD..QL 493**

**SRK45 446:.SSTVA...-.L.LIM----..L.K..Q.R...S..S.ANRQR..N.SMNGMVLLS..E. 500**

**SRK29 508:SGEDEVENLELPLMEFEAVVTATEHFSDFNKVGKGGFGVVYKGRLVDGQEIAVKRLSEMS 567**

**SRK40 509:...........S................................................ 568**

**SRK44 508:............................L............................... 567**

**SRK60 509:...E....F................................................... 567**

**SRK8 505:.R.NKADEF....I.L....K...N..NC.EL.R....I....-ML....V......KT. 563**

**SRK12 503:.R.NKT.EF....I.L....K...N..NC.EL.Q....I....-ML....V......KT. 561**

**SRK21 504:PR.NKI.E.....I.L....K...N..NC..L.Q....I......L...........KT. 563**

**SRK25 488:P..KKI.E.....I.L.T..K...N...C..L.Q....I......L...........KT. 547**

**SRK35 494:.R.NKADEF....I.L....K...N..NC.EL.R....I....-ML....V......KT. 552**

**SRK45 501:.VKNKI.E.....I.L....K..DN..NC..L.Q....I......L............T. 560**

**SRK29 568:AQGTDEFMNEVRLIAKLQHNNLVRLLGCCVYEGEKILIYEYLENLSLDSHLFDGSRSCKL 627**

**SRK40 569:.....................................................ET...M. 628**

**SRK44 568:.....................................................ET...M. 627**

**SRK60 569:S........................................M...........ET.G.M. 628**

**SRK8 564:L..I...........R...I....I....IEA............S...YF..GKK..SN. 623**

**SRK12 562:L..I...........R...I....I....IEAD...........S...YF..GKK..SN. 621**

**SRK21 564:V..............R...I....I....IDA..TM........S....Y..GKK..... 623**

**SRK25 548:V..........T...R...I...QII...IEAD..M............CY..GKTQRS.. 607**

**SRK35 553:L..I...........R...I....I....IEAD...........S...YF..GKK..SN. 612**

**SRK45 561:V..........T...R...I...QI....IEAD..M.............Y..GKTQRS.. 620**

**SRK29 628:NWQMRFDIINGIARGLLYLHQDSRFRIIHRDLKASNVLLDKDMTPKISDFGMARIFGRDE 687**

**SRK40 629:.........................................................Q.. 688**

**SRK44 628:.........S.................................................. 687**

**SRK60 629:............................................................ 688**

**SRK8 624:..KD..A.T..V.....................PG.I....Y.I............A... 683**

**SRK12 622:..KD..A.T..V.....................PG.I....Y.I............A... 681**

**SRK21 624:..KD....T..V...................M.V..I...QN.I...........VA... 683**

**SRK25 608:..KE.......V.....................V..I....N.I............A... 667**

**SRK35 613:..KD..A.T..V.....................PG.I....Y.I............A... 672**

**SRK45 621:..KE....T..V.....................V..I....N.I............A... 680**

**SRK29 688:TEADTRKVVGTYGYMSPEYAMNGTFSMKSDVFSFGVLLLEIISGKRNKGFCDSDSSLNLL 747**

**SRK40 689:............SIKHL......................................N.... 748**

**SRK44 688:.......................................................T.... 747**

**SRK60 689:............................................................ 748**

**SRK8 684:.QVR.DNA.............Y.VI.E.T.......IV...VI....R..YQVNPEN..P 743**

**SRK12 682:IQ.R.DNA.............D.VI.E.T.......IV...V.....R..YQVNPEN..P 741**

**SRK21 684:...N..N..............D.V..E.........IV........SR..YHLNHEN... 743**

**SRK25 668:...N.M...............R.I..E.........IV...V...K.SR.YKLNCEND.. 727**

**SRK35 673:.QVR.DNA.............Y.VI.E.T.......IV...VI....R..YQVNPEN..P 732**

**SRK45 681:...N.M.................I..E.........IV...VT....-RGY------.F. 733**

**SRK29 748:GCVWRNWKEGQGLEIVDRVIIDS---SSPTFRPSEISRCLQIGLLCVQERVEDRPMMSSV 804**

**SRK40 749:.......................---.......R..L....................... 805**

**SRK44 748:.................KF.N..---.....K.R..L....................... 804**

**SRK60 749:.......................---.................................. 805**

**SRK8 744:SYA.TH.A..RA.....P..L..LSSLPS..K.K.VLK.I......I...A.H..T.... 803**

**SRK12 742:SY..TH.A..RA.....P..L..LSSLPS..K.K.VLK.I......I...A.H..T.... 801**

**SRK21 744:SY..SH.T..RA.....P..V..LSSLAA..Q.K.VLK.I..........A.H..T.... 803**

**SRK25 728:SYA.SH....RA.....P..V..LPSLPL.SQ.Q.VLK.I..........A.H..T.A.. 787**

**SRK35 733:SYA.TH.A..RA.....P..L..LSSLPS..K.K.VLK.I......I...A.H..T.... 792**

**SRK45 734:SYA.SH....RT..L..P..V..--SLPS..Q.E.VLK.I.........LA.H..T.... 791**

**SRK29 805:VLMLGSEAALIPQPKQPGYCVSGSSLETY---SRR--DDENWTVNQITMSIIDAR 854**

**SRK40 806:.......T..............Q......---...--.................. 855**

**SRK44 805:.............................---...--.............N.... 854**

**SRK60 806:.............................---...--.................. 855**

**SRK8 804:.W......TE.....P.V..LIA.YYANNPSS..QFD...S....KY.C.V.... 858**

**SRK12 802:.W......TE.....P.V..LIA.YYANNPSS..QFD...S....KY.C.V.... 857**

**SRK21 804:.R......TE.....P....LVS.HY.NNPSS..YCN...S.....Y.C.V.... 858**

**SRK25 788:.W......TD.....P....IQR.PY.LDPSS..QCNE..S.....Y.C.L.... 842**

**SRK35 793:.W......TE.....P.V..LIA.YYANNPSS..QFD...S....KY.C.V.... 847**

**SRK45 792:.W......TE..H..P..C.IGR.PY.LEPSS..QCDE..S.....Y.C.V.... 846**

**Supplementary Fig. S1**

Sequence alignment of SRK cytoplasmic region. The amino acid sequences of the transmembrane and cytoplasmic domains of 6 class-I SRKs and 4 class-II SRKs in *B. rapa* were aligned. The SRK^29^-specific amino acid site in class-II SRKs is shown in red. Identical amino acids are represented by dots. Black, green, red, and blue lines represent the transmembrane domain, DUF3660, kinase domain, and DUF3403, respectively.
